# EPIP-Evoked Modifications of Redox, Lipid, and Pectin Homeostasis in the Abscission Zone of Lupine Flowers

**DOI:** 10.3390/ijms22063001

**Published:** 2021-03-16

**Authors:** Emilia Wilmowicz, Agata Kućko, Wojciech Pokora, Małgorzata Kapusta, Katarzyna Jasieniecka-Gazarkiewicz, Timothy John Tranbarger, Magdalena Wolska, Katarzyna Panek

**Affiliations:** 1 Department of Plant Physiology and Biotechnology, Nicolaus Copernicus University, 1 Lwowska Street, 87-100 Toruń, Poland; magda_w@umk.pl (M.W.); ahafija87@gmail.com (K.P.); 2 Department of Plant Physiology, Institute of Biology, Warsaw University of Life Sciences-SGGW (WULS-SGGW), Nowoursynowska 159 Street, 02-776 Warsaw, Poland; agata_kucko@sggw.edu.pl; 3 Department of Plant Physiology and Biotechnology, University of Gdańsk, 59 Wita Stwosza, 80-308 Gdańsk, Poland; wojciech.pokora@biol.ug.edu.pl; 4 Department of Plant Cytology and Embryology, University of Gdańsk, 59 Wita Stwosza, 80-308 Gdańsk, Poland; malgorzata.kapusta@ug.edu.pl; 5 Intercollegiate Faculty of Biotechnology of University of Gdansk and Medical University of Gdańsk, Abrahama 58, 80-307 Gdańsk, Poland; katarzyna.jasieniecka@biotech.ug.edu.pl; 6 UMR DIADE, IRD Centre de Montpellier, Institut de Recherche pour le Développement, Université de Montpellier, 911 Avenue Agropolis BP 64501, 34394 CEDEX 5 Montpellier, France; timothy.tranbarger@ird.fr

**Keywords:** cell wall, crops, fatty acids, flower abscission zone, oxidative stress, yellow lupine

## Abstract

Yellow lupine is a great model for abscission-related research given that excessive flower abortion reduces its yield. It has been previously shown that the EPIP peptide, a fragment of LlIDL (INFLORESCENCE DEFICIENT IN ABSCISSION) amino-acid sequence, is a sufficient molecule to induce flower abortion, however, the question remains: What are the exact changes evoked by this peptide locally in abscission zone (AZ) cells? Therefore, we used EPIP peptide to monitor specific modifications accompanied by early steps of flower abscission directly in the AZ. EPIP stimulates the downstream elements of the pathway—*HAESA* and MITOGEN-ACTIVATED PROTEIN KINASE6 and induces cellular symptoms indicating AZ activation. The EPIP treatment disrupts redox homeostasis, involving the accumulation of H_2_O_2_ and upregulation of the enzymatic antioxidant system including superoxide dismutase, catalase, and ascorbate peroxidase. A weakening of the cell wall structure in response to EPIP is reflected by pectin demethylation, while a changing pattern of fatty acids and acyl lipids composition suggests a modification of lipid metabolism. Notably, the formation of a signaling molecule—phosphatidic acid is induced locally in EPIP-treated AZ. Collectively, all these changes indicate the switching of several metabolic and signaling pathways directly in the AZ in response to EPIP, which inevitably leads to flower abscission.

## 1. Introduction

Organ separation from the plant body is a physiological process and a fundamental mechanism that allows plants to adapt to unfavorable environmental conditions and ensure reproductive success. However, premature and excessive abscission of generative organs reduces crop yield quantity and quality, thus causing serious economic losses. Activation of specialized cells that constitute an abscission zone (AZ), usually located at the base of an organ, is required to induce organ detachment [1,2,3,4]. AZ functioning is a very complex and highly coordinated process regulated by the interdependent action of many molecular and biochemical factors. The synchronous action of pathways induced by these factors leads to the specific structural changes of AZ cells, degradation of the middle lamella, disruption of cell-to-cell adhesion, and finally organ abscission. Elucidation of the elements of this complex machinery is of great importance for basic studies, but it could also provide solutions for the major agronomic challenges related to organ detachment. Over the past years, several papers focused on organ abscission in crop species, e.g., *Litchi chinensis*, citrus, *Populus tremula*, *Glycine max*, *Elaeis guineensis*, and *Solanum lycopersicum*, have been published [5,6,7,8]. Among crops, a great model for such research is *Lupinus luteus*, in which premature and excessive abortion of flowers occurs [9,10,11,12,13]. However, the regulatory mechanisms of plant organ abscission have been investigated, mainly in the model plant *Arabidopsis* [14,15,16,17,18]. In that species, floral organ abscission involves a peptide–receptor signaling pathway that was identified to function. The pathway consists of the INFLORESCENCE DEFICIENT IN ABSCISSION (IDA) peptide, HAESA/HAESA-LIKE2 (HAE/HSL2) receptors, and MITOGEN-ACTIVATED PROTEIN KINASE (MKK4/MKK5 and MPK6) signaling cascade [19,20,21]. It has been shown that the IDA peptide, which is a ligand for the transmembrane receptor-like kinases (RLKs) HAE/HSL2, comprises a signaling pathway (IDA–HAE–HSL2) that activates abscission processes [6,8,14,16]. The amino acid sequence of IDA contains a highly conserved, C-terminal EPIP (extended PIP) domain that is critical for signaling activity [14,16,22]. A proline localized in the EPIP domain binds to the extracellular part of the HAE/HSL2 receptor and causes its autophosphorylation. This, in turn, triggers in the cytoplasm of AZ cells a signal transduction pathway involving MAP kinases that activates transcriptional factors, e.g., KNAT2 and KNAT6 (knotted-like from *Arabidopsis thaliana* 2 and 6), which induce organ abscission [23,24]. AZ activation requires intensive de novo synthesis of proteins [25] that are involved in the degradation of cell walls, but also in some cases the formation of newly-formed cell walls of daughter cells. Moreover, observations of cell organelles, including chloroplasts and nuclei, indicated DNA fragmentation and possible induction of programmed cell death (PCD), probably caused by intensive reactive oxygen species (ROS) generation [2,20,23,24,26]. The involvement of ROS in the functioning of AZ cells and organ abscission has been demonstrated in many plant species [26,27,28]. Hydrogen peroxide (H_2_O_2_) has been shown to induce the expression of the cell wall-degrading enzymes at the execution phase of abscission [27]. ROS produced extensively in the AZ can be responsible for pathological effects in different subcellular compartments, and they can damage lipid bilayers due to their contribution to the degradation of fatty acids released from cell membranes [29,30].

Homologs of *IDA*/*IDL* genes were found in many plant species, e.g., *G. max*, *S. lycopersicum*, *L. luteus*, *P. tremula*, *E. guineensis*, and citrus (sweet orange and clementine) [5,7,10,31]. Gene expression analyses, experiments on transgenic plants, and the use of synthetic peptides (EPIPs or PIPs) obtained based on IDA or IDL sequences, provides evidence that the abscission mechanism is governed by IDA and may have common features both in monocots and dicots [7,8,31]. *LcIDL1* is an abscission-associated gene expressed during the male flower and fruitlet drop in *L. chinensis* [7]. Furthermore, citrus *CitIDA3* functions to promote floral organ abscission in transgenic *Arabidopsis*, and ectopic expression of *CitIDA3* could also complement the abscission deficiency of the *ida* mutant [6]. IDA and IDL1 EPIP synthetic peptides have been reported to rescue *ida* and induce early floral abscission of wild-type *A. thaliana* flowers [16]. Our analyses performed in vivo on *L. luteus* have shown that exogenous EPIP peptide stimulated the abortion of flowers [10]. Moreover, synthetic PIPs can enhance dark-induced leaf abscission in *P. tremula* and ripe fruit abscission *E. guineensis* [8]. Although it is known that EPIP determines IDA/IDL protein activity, and it is certainly sufficient to initiate a plant response [16,32], and there is no report that aimed to monitor subsequent specific cellular changes evoked by this peptide. Therefore, a fundamental goal is to elucidate the exact consequences of EPIP action at the physiological level and to determine which abscission-related events are induced by EPIP locally in AZ cells. We can now address these questions in *L. luteus* given that we have already investigated the anatomical, biochemical, and physiological features that accompany the initial stages of activation of abscission, as well as those related to the natural separation processes [10]. Thus, treatments with the synthetic EPIP provides an experimental approach to follow the initial stages of the mechanisms that activate and induce cell separation events in the flower AZ.

In the presented study, we made use of the bioactive potential of synthetic EPIP peptide to monitor cellular, molecular, and biochemical AZ-specific modifications in the flower of yellow lupine evoked after EPIP-induced abscission. We examined the peptide influence on the downstream elements of the LlIDA pathway, redox homeostasis, lipid metabolism, and cell wall components. Collectively, all observed changes are a manifestation of the induction of metabolic and signaling pathways in the AZ, which activates this structure and leads to flower abscission.

## 2. Results

### 2.1. EPIP Peptide Induces Subsequent Components of a Pathway Responsible for Flower AZ Activation

**Figure 1:**
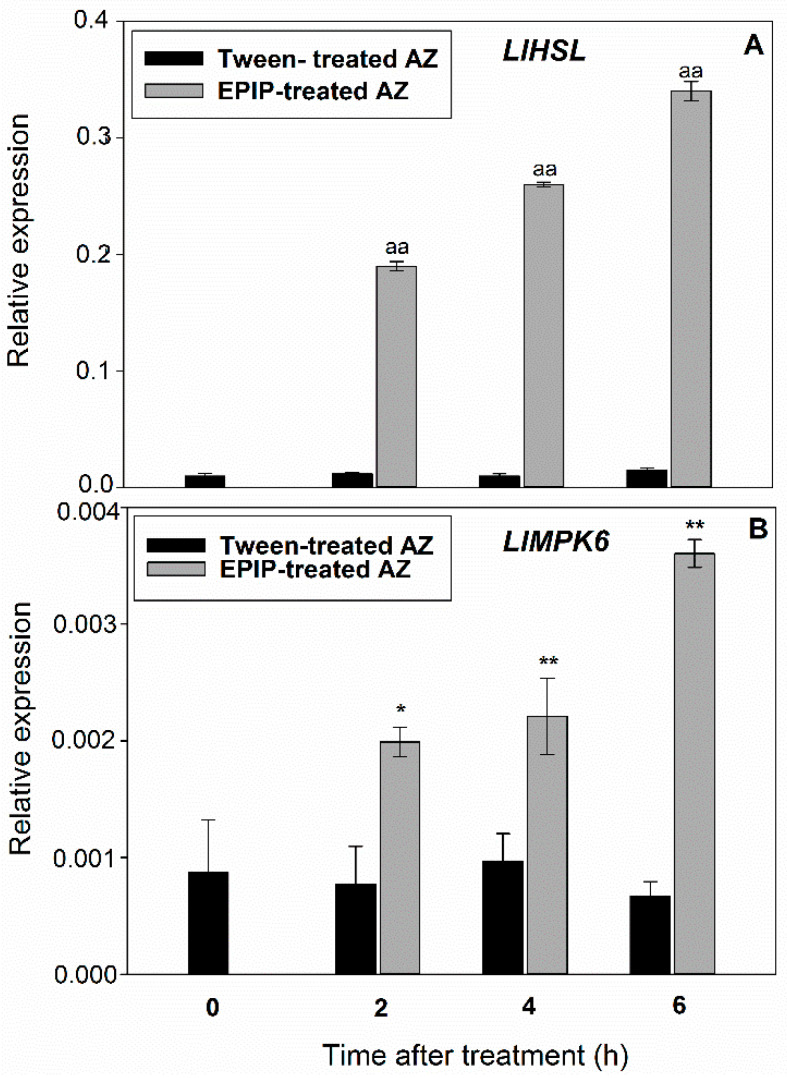
Exogenous EPIP affects molecular elements of the abscission-related signaling pathway in the floral abscission zone (AZ) of yellow lupine. Transcriptional activity of *LlHSL* (**A**), and *LlMPK6* (**B**) (related to *LlACT*) in AZ after EPIP treatment. Fragments of AZ were excised at 2 h, 4 h, and 6 h after EPIP (100 µM) solution in 0.05% Tween 20 applied directly on inactive AZ. The control was inactive AZ dissected at the same time variants from the flower bases after the application of the 0.05% Tween 20 solution. Data are presented as averages ± SE. For *LlHSL*, significant differences for EPIP treated plants in comparison to control are indicated as ^aa^ *p* < 0.01; for *LlMPK6*, significant differences for EPIP treated plants in comparison to control are indicated as ** *p* < 0.05, * *p* < 0.01.

Our previous experiments showed that flower AZ activation in *L. luteus* is associated with the upregulation of genes encoding elements of the molecular abscission-associated pathway (*LlIDL*, *LlHSL*, and *LlMPK6*) [10,11]. We have also experimentally established that the synthetic EPIP peptide, obtained from the LlIDL sequence, applied directly to inactive AZ tissue, stimulated the separation of yellow lupine flowers [10]. In the presented paper, we used the already verified experimental system to investigate what molecular, biochemical, and cellular events are evoked by exogenous EPIP, and, as a consequence, flower separation. We first confirmed that *LlHSL* is rapidly stimulated in response to EPIP applied directly on inactive flower AZ (Figure 1A). By 2 h after exogenous EPIP treatment, the *LlHSL* transcript accumulated approximately 20 times higher compared to inactive AZ (control), while the highest value was observed 6 h after exogenous EPIP treatment. Similarly, the peptide treatment led to a gradual increase of *LlMPK6* mRNA (Figure 1B). At 2 h after treatment, *LlMPK6* transcripts accumulated twice as much as in inactive AZ, remained high at 4 h, and reached a peak of 4 times higher than the control at 6 h.

**Figure 2:**
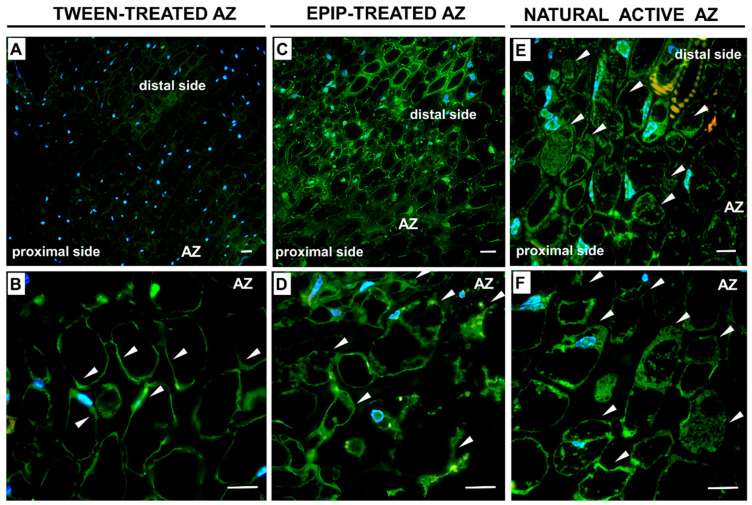
Immunolocalization of MPK6 in the flower AZ of yellow lupine in response to EPIP treatment and the naturally active AZ. EPIP peptide solution (100 µM) in 0.05% Tween 20 was applied directly on inactive AZ and the plant material was collected 6 h after treatment (**C**,**D**). Inactive AZ was harvested 6 h after 0.05% Tween 20 solution application (**A**,**B**). MPK6 was also localized in the naturally active AZ (**E**,**F**). Photos **B**, **D**, and **F** are magnifications of AZ regions used for analyses presented on **A**, **C**, and **E**. Bar—60 µm (**A**), 100 µm (**B**), 40 µm (**C**–**F**). Immunofluorescence, green signal indicates MPK6 presence. Nuclei were stained with DAPI (blue fluorescence). Arrows mark signal for MPK6 in the peripheral areas of the cytoplasm (**B**) and in the cell cytosol after cell division (**D**–**F**). Yellow and orange color corresponds to the cell wall autofluorescence.

The time-variant, at which *LlMPK6* reached the highest values (6 h), was chosen for the immunofluorescence experiments. We detected a strong accumulation of MPK6 in the distal and proximal areas adjacent to the AZ treated with EPIP (Figure 2C). The fluorescence signal forms clusters occurring in the place of newly forming cell walls, as well as in the peripheral areas of the cytoplasm (Figure 2D). In the inactive AZ, the signal is weak, and we only observed MPK6 in the bordering areas of the cytoplasm (Figure 2A,B). In turn, labeling is observed in the cytoplasm and the place of new cell walls formed after cell proliferation in naturally active AZ (Figure 2E,F). The distribution of MPK6 in EPIP-treated AZ (Figure 2D) is similar to that observed in the naturally active structure (Figure 2F). Control reaction with primary antibody omission gave negative results (Supplementary Figure S1B). Collectively, these results provide evidence that the EPIP treatment increases MPK6, a signaling component acting downstream of the IDA–HAE–HSL2 pathway in the flower AZ.

### 2.2. The Synthetic EPIP Peptide Treatment Results in Cellular Changes in the Floral AZ Similar to Those That Occur in the Naturally Active AZ

**Figure 3:**
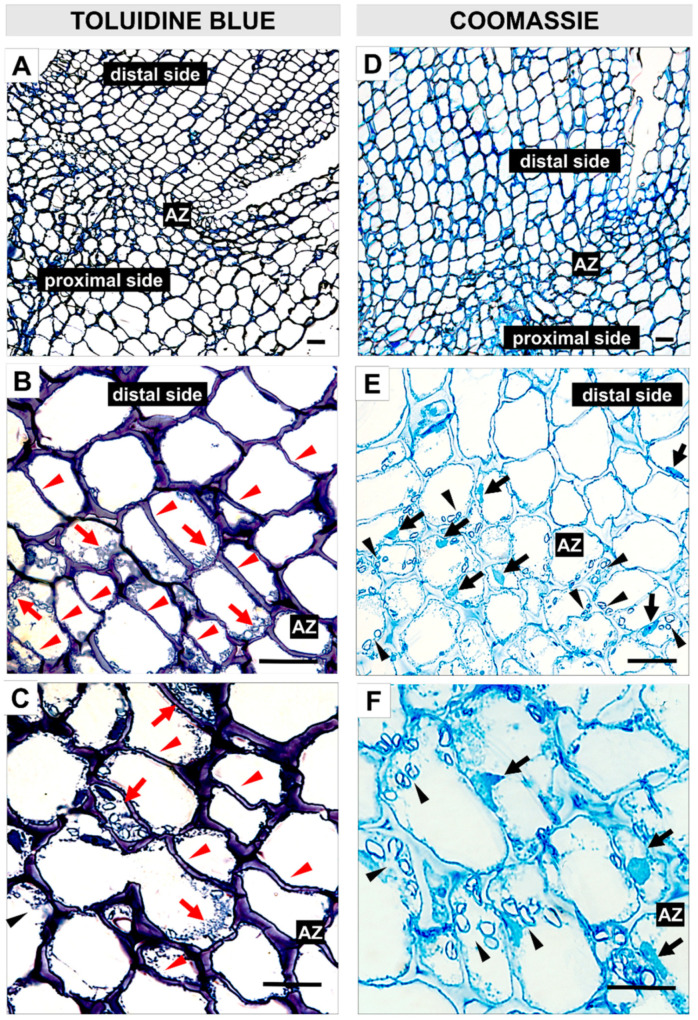
Synthetic EPIP peptide evokes specific cellular changes in the flower abscission zone (AZ) of *L. luteus*. A 100 µM peptide solution in 0.05% Tween 20 was applied directly to the inactive AZ. For histological observations, sections of AZ were collected 24 h after the EPIP application. Fixed material was stained with toluidine blue (**A**–**C**) or Coomassie Brilliant Blue (**D**–**F**). Pictures **B**, **C**, and **E**, **F** are magnifications of different areas presented in **A** and **D**, respectively. Enlarged AZ cells (**C**,**F**). Red arrows indicate aggregates and small vesicles, while red arrowheads mark the place of newly formed cell walls after divisions (**B**,**C**). Black arrows indicate elongated, protein-rich cell nuclei, while black arrowheads correspond to the presence of aggregates and small vesicles enriched in proteins (**E**,**F**). Bar—40 µm.

In the current study, we performed experiments to elucidate the influence of EPIP on AZ-specific cellular changes. Firstly, the EPIP was applied to the AZ of non-abscising flowers and 24 h after treatment intensive cell divisions in the AZ area were noticed (Figure 3A–C). At the same time, inactive AZ from non-abscising flowers was treated with 0.05% Tween 20 for 24 h (Supplementary Figure S2). In the EPIP-treated AZ, we observed newly synthesized cell walls of recently divided cells. These cells have numerous narrow regions across the cell walls, visible cellular aggregates, and small vesicles (Figure 3B,C). In addition, the Coomassie Brilliant Blue staining revealed an abundance of proteins (Figure 3E,F). The cells located in the distal and proximal parts of AZ are different in size and shape compared to those in the EPIP-treated AZ layer (Figure 3A,D). In contrast to EPIP-activated AZ cells, inactive AZ contains round, non-dividing, loosely arranged cells (see Supplementary Figure S2).

### 2.3. EPIP Peptide Influences the Redox Homeostasis in Flower AZ

**Figure 4:**
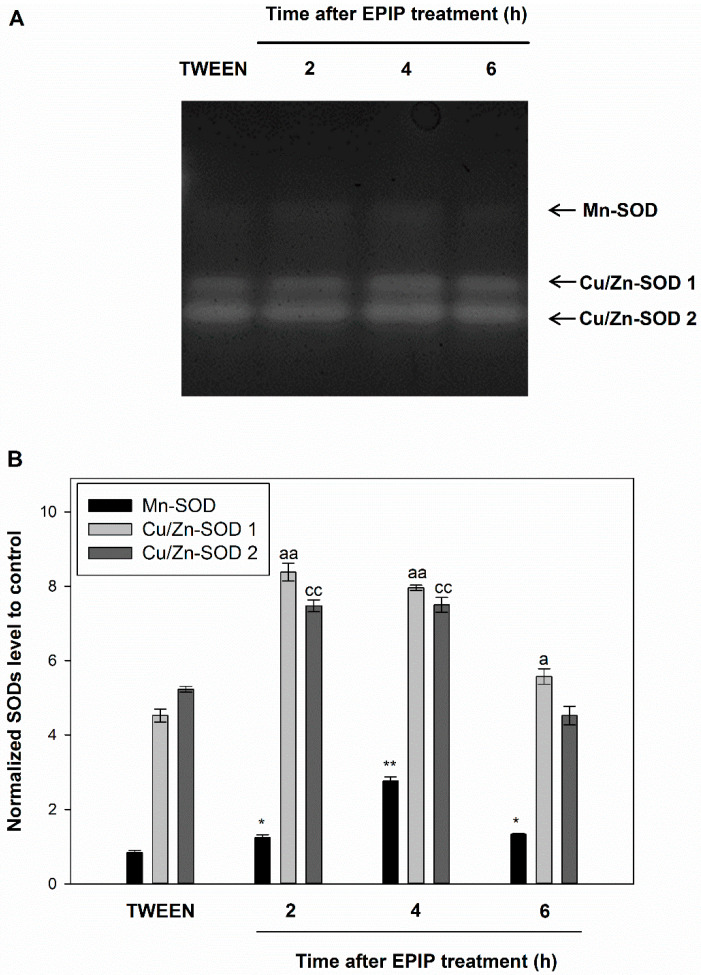
Exogenous EPIP affects the enzymatic activity of superoxide dismutase (SOD) in the floral abscission zone (AZ) of yellow lupine. SOD isoenzyme profile was obtained for fragments of AZ excised at 2 h, 4 h, and 6 h after EPIP (100 µM) solution in 0.05% Tween 20 treatment. EPIP was applied directly to inactive AZ. The inactive AZ was dissected at the same time variants from the flower bases after the application of the 0.05% Tween 20 solution. Mn-SOD and two isoforms of Cu/Zn-SOD were detected using an in-gel assay. Representative micrograph of NBT-stained gel is presented (**A**). The chart (**B**) displays the average densitometry data corresponding to the bands detected on three separate gels. Each band was quantified and expressed as value compared to inactive AZ. The values for each isoform were normalized to the inactive AZ after Tween 20 application. Data are presented as averages ± SE. For Mn-SOD, significant differences for EPIP treated plants in comparison to control are indicated as * *p* < 0.05, ** *p* < 0.01; for Cu/Zn-SOD1, significant differences for EPIP treated plants in comparison to control are indicated as ^a^ *p* < 0.05, ^aa^ *p* < 0.01; for Cu/Zn-SOD2, significant differences for EPIP treated plants in comparison to control are indicated as ^cc^ *p* < 0.01.

Our previous research indicates that activation of the flower AZ in yellow lupine leads to the accumulation of ROS, including H_2_O_2_ [11,12]. The accumulation of ROS is associated with an increase in catalase (CAT) activity [11]. In the current study, we aimed to determine whether EPIP evokes changes in the ROS balance in AZ cells and influences the activity of ROS-detoxifying enzymes, including superoxide dismutase (SOD), responsible for superoxide detoxification, CAT, and ascorbate peroxidase (APX), which oxidizes H_2_O_2_ [33,34]. We observed that different SOD isoforms extracted from the inactive flower AZ are active (Figure 4A). One of them requires manganese (Mn-SOD) as a cofactor and is responsible for mitochondrial ROS accumulation [35], while two copper-zinc (Cu/Zn-SOD1, Cu/Zn-SOD2) isoforms are localized in chloroplasts [33]. Mn-SOD shows the lowest enzymatic activity among the SODs analyzed in all tested variants. The enzymatic activity of Cu/Zn-SOD1 and Cu/Zn-SOD2 in inactive AZ is similar, over 5 times higher than Mn-SOD (Figure 4B). The EPIP treatment accelerates the enzymatic activity of all SODs. The greatest increase is observed for Mn-SOD (4 h after EPIP treatment), at almost three times higher than in inactive AZ (Figure 4B). However, the highest activity was observed for Cu/Zn-SOD1, which within 2 h after EPIP application, had an almost 50% higher activity than in the inactive control AZ. Similarly, the Cu/Zn-SOD2 enzymatic activity was higher at both 2 h and 4 h after EPIP treatment.

**Figure 5:**
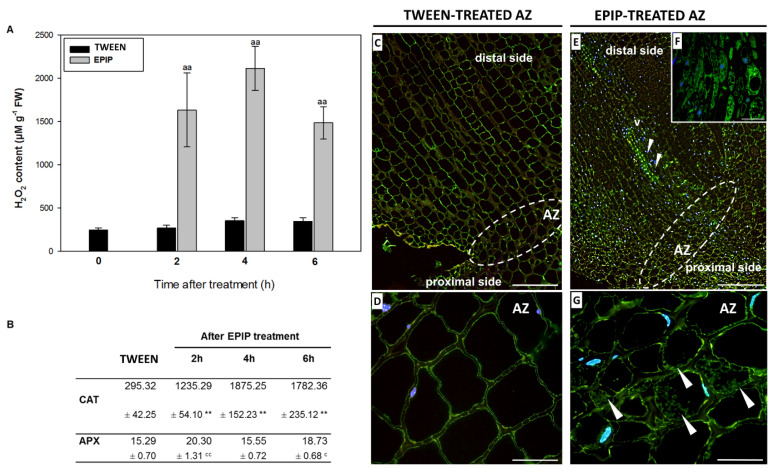
Exogenous EPIP stimulates the hydrogen peroxide (H_2_O_2_) formation (**A**), accelerates the enzymatic activity of catalase (CAT) and ascorbate peroxidase (APX) (**B**), and changes CAT localization (**C**–**G**) in floral abscission zone (AZ) of yellow lupine. The EPIP peptide (100 µM in 0,05% Tween 20) was applied directly to inactive AZ. The AZ samples were excised after 2 h, 4 h, and 6 h of treatment. The values for each isoform were normalized to the inactive AZ treated 0.05% Tween 20 solution. Data are presented as averages ± SE. For H_2_O_2_, significant differences for EPIP treated plants in comparison to Tween-treated are indicated as ^aa^ *p* < 0.01; for CAT, significant differences for EPIP treated plants in comparison to Tween-treated are indicated as ** *p* < 0.01, for APX significant differences for EPIP treated plants in comparison to control are indicated as ^c^ *p* < 0.05, ^cc^ *p* < 0.01. The localization of CAT was examined 6 h after the EPIP application. The green signal marked by arrows corresponds to CAT presence, while blue fluorescence to DAPI-stained nuclei. White lines were used to mark AZ regions. Bars—100 µm (**A**,**E**), 40 µm (**D**,**G**), 20 µm (**F**).

The increase in SOD enzymatic activity after EPIP application corresponds to the accumulation of H_2_O_2_ in AZ (Figure 5A). Two hours after EPIP treatment, the H_2_O_2_ amounts are over 5 times higher than in inactive AZ. The highest value is observed at 4 h of EPIP-treated AZ, while the value decreased at 6 h, yet remains higher than values in the inactive AZ. Oxidative stress conditions trigger the enzymatic antioxidant system. Indeed, the increase in H_2_O_2_ consequently elevates CAT activity at 2 h, 4 h, and 6 h, and APX activity at 2 h and 6 h significantly in AZ cells after EPIP treatment (Figure 5B). EPIP also affects tissue and cellular localization of CAT (Figure 5E–G). We observe a strong fluorescence signal indicating the enzyme presence in vascular bundles of the whole pedicel (Figure 5E,F) and neighboring cells (Figure 5F). CAT is also localized in the cytoplasm of AZ cells treated with EPIP (Figure 5G), while lower amounts of this enzyme is present in inactive AZ (Figure 5C,D). Obtained results indicate that the EPIP treatment strongly disturbs redox balance in AZ cells.

### 2.4. Exogenous EPIP Results in Enhanced Lipid Content and Changes Their Composition in AZ Cells

**Figure 6:**
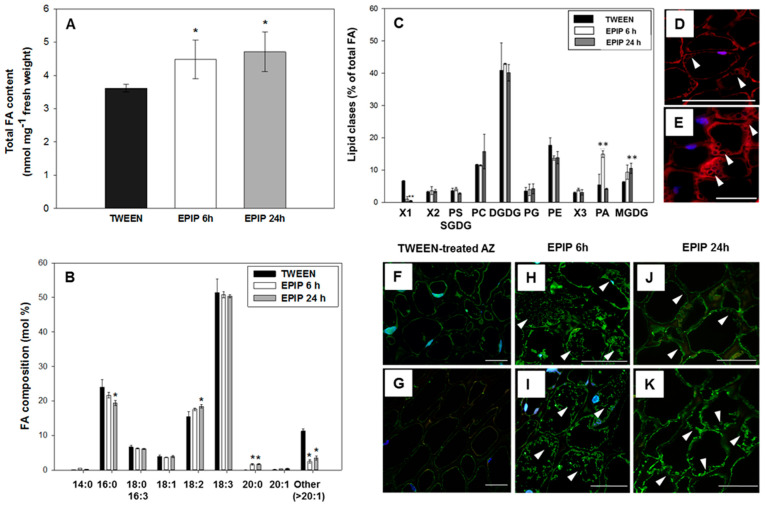
EPIP strongly influences lipid homeostasis in the floral abscission zone (AZ) of yellow lupine. Total fatty acid (FA) content (**A**) and composition (**B**), composition of all acyl-lipids (**C**), neutral lipids presence (**D**,**E**), and phospholipase D (PLD) localization (**F**–**K**). The fragments of AZ were excised on the 6 h and 24 h after application of EPIP (100 µM) solution in 0.05% Tween 20 directly to inactive AZ. Inactive AZ was dissected from the flower bases after the application of the 0.05% Tween 20 solution only. Error bars on the charts indicate SE. For total FA content in acyl-lipids, significant differences for EPIP-treated plants in comparison to Tween-treated are indicated as * *p* < 0.05 (**A**). For FA composition, significant differences for EPIP treated plants in comparison to control are indicated as * *p* < 0.05 (**B**). For lipid classes significant differences for EPIP treated plants in comparison to Tween-treated are indicated as ** *p* < 0.01 (**C**). Abbreviations: PS—phosphatidylserine, SQDG—sulfoquinovosyl-diacylglycerol, PC—phospholipids phosphatidyl-choline, DGDG—digalactosyl-diacylglycerol, PG—phosphatidylglycerol, PE—phosphatidyl-ethanolamine, PA—phosphatidic acid, MGDG—monogalactosyl-diacylglycerol, X1, X2, and X3—unidentified lipids localized on the TLC plate. The localization of neutral lipids (red fluorescence marked by arrows) was analyzed using Nile Red staining of sections dissected 6 h after EPIP treatment (**D**,**E**). PLD was localized 6 h (**H**,**I**) and 24 h (**J**,**K**) after EPIP application, as well as in Tween-treated AZ (**F**,**G**). Photos G, I, and K are magnifications of F, H, and J, respectively. The green fluorescence marked by arrows corresponds to PLD presence. Nuclei were stained with DAPI (blue fluorescence). Bars—50 µm (**D**,**E**), 60 µm (**F**–**K**).

Accumulated ROS can lead to oxidative degradation of lipids, which are the main components of cell membranes [36,37,38,39] that are supposed to play a significant role in cell stability, cell-to-cell adhesion, and organ separation. Moreover, lipids and fatty acids (FAs) may also be signaling molecules and/or precursors of other molecules related to abscission, e.g., phytohormones, like ABA or JAs [36]. Studies on the involvement of lipids in separation processes are limited, but their roles during abscission and stress responses have been suggested in previous studies [39,40]. Transcriptome analyses indicate that genes associated with lipid metabolism are expressed specifically in AZ cells [40]. In the current study, histological analysis of the flower AZ cells of lupine suggests the appearance of numerous vesicular structures in response to exogenous EPIP (Figure 2B,C). The presence of vesicles could be associated with lipid synthesis and/or transport [41]. Therefore, we examined the effect of the EPIP peptide on lipid changes in floral AZ. Exogenous EPIP increases the total FA content of acyl lipids after both 6 h and 24 h of peptide application (Figure 6A). The observed effect may be associated with strong cell divisions characteristic for EPIP-activated AZ (Figure 3B,C) and the synthesis of lipids necessary for the formation of membranes of daughter cells. Nile red staining revealed the localization of structures enriched in acyl lipids in the cells of floral AZ treated with EPIP (Figure 6D,E). In addition, strong labeling was emitted by the cell membranes of dividing AZ cells (Figure 6D). It has long been known that FAs composition of membrane lipids changes depending on environmental conditions. Their composition is important for determining the membrane lipid fluidity and the ability of plants to react against stresses [42]. Fatty acid composition of membrane lipids is also critical for preventing the damages evoked by ROS, thus they could also mediate abscission-associated processes. The EPIP peptide treatment also affects the composition of fatty acid acyl-lipids in the AZ (Figure 6B). A decrease in the content of palmitic acid (16:0), and an increase in linoleic acid (18:2) at 24 h after EPIP application was observed. Arachidic acid (20:0) content increased at both 6 h and 24 h after the EPIP application (Figure 6B).

The lipid composition of the membrane can also affect its properties and thus the function of the whole cells. We next used TLC and GC analyses to determine the changes in lipid composition following EPIP application to the floral AZ. The analysis revealed changes in the lipid profile in floral AZ cells (Figure 6C), such as an increase in phosphatidic acid (PA) and monogalactosyldiacylglycerol (MGDG) at 6 h and 24 h after treatment, respectively. By using additional immunofluorescence techniques, we observed a positive correlation between the high PA level in the AZ and the appearance of PLD (phospholipase D; Figure 6H,I), an enzyme that catalyzes PA formation via degradation of other phospholipids. PLD accumulated more at 6 h after EPIP treatment (Figure 6H,I) when compared to the subsequent time-variant (24 h) (Figure 6J,K) and to the Tween-treated AZ (Figure 6F,G). It is well known that between ER and plastids, a bulk transfer of lipids is occurring [43,44]. The strong increase of the relative amount of PA at 6 h after EPIP treatment and the decrease of PA quantities after that time, with a simultaneous increase in the relative amount of MGDG, suggests that PA could be transferred to the plastid and serve as a substrate for MGDG synthesis. Prior to PA’s use for MGDG synthesis, it must be converted to diacylglycerol (DAG) via phosphatidic acid phosphatase action, an enzyme presents both in the ER and inner chloroplast membrane [43]. Thus, PA can be transferred to the plastid both as a PA molecule or as DAG molecule. In the flower AZ of *L. luteus*, relatively low quantities of phosphatidylserine (PS), SQDG, and PG were observed and did not change under the influence of EPIP peptide (Figure 6C). The EPIP-treated AZ structure has a higher level of phosphatidylcholine (PC) and phosphatidylethanolamine (PE), and a very high amount of digalactosyl-diacylglycerol (DGDG) when compared to other acyl lipids. However, none of these compounds changed significantly in response to the EPIP treatment.

### 2.5. EPIP-Induced Flower Abscission Is Accompanied by Concomitant Modifications of Pectin Cell Wall Components

Homogalacturonans (HGs) are the main polymer of the pectin rich middle lamella [45,46]. Homogalacturonans are synthesized de novo and integrated into the cell wall in a highly methyl-esterified form, which makes HG relatively fluid, while de-methyl-esterification of HG by pectin methyl-esterases (PME, EC 3.1.1.11) results in a more elastic cell wall [47]. Considering these facts, we examined the effect of EPIP on the degree of pectin methylesterification in flower AZ. We used the JIM5 antibody that recognizes low methylesterfied or un-methylesterfied forms of HG, and the JIM7 antibody, which recognizes higher-order methylesterified HG than JIM5 [48,49]. We used inactive, Tween-treated AZ, as well as EPIP-treated AZ, and naturally active AZ to compare pectin distribution in different circumstances and to verify whether EPIP can evoke cell wall remodeling, characteristic for abscission. The control reaction was performed, with the primary antibody omitted (see Supplementary Figure S1).

**Figure 7:**
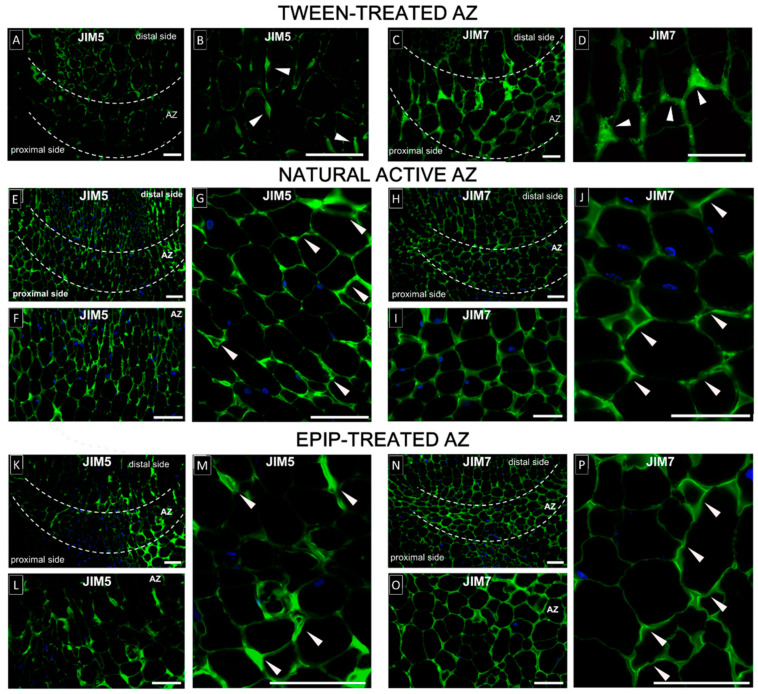
EPIP affects pectin cell wall composition in the floral AZ. Immunofluorescence localization of methylesterified pectin was performed in the yellow lupine flower abscission zone (AZ). Green fluorescence indicates low methylated and unmethylated HGs (JIM5 labeling **A**,**B**,**E**–**G**,**K**–**M**) and high methylated HGs (JIM7 labeling, **C**,**D**,**H**–**J**,**N**,**O**,**P**). Immunofluorescence reactions were performed for the Tween-treated AZ (**A**–**D**), naturally active AZ (**E**–**J**), and EPIP-treated AZ (**K**–**P**). The 0.05% Tween 20 or EPIP peptide solution (100 µM in 0.05% Tween 20) were applied directly to inactive AZ. The plant material was collected 6 h after each treatment (**A**–**D**,**K**–**P**). Naturally active AZ was excised from abscised flowers (**E**–**J**). Fluorescence signal corresponding to pectin presence is indicated by arrows. Nuclei were stained with DAPI (blue fluorescence). AZ regions are marked by white, dotted lines. Bar—50 µm.

The JIM5 signal is detected in the walls of proliferated cells of naturally active AZ (Figure 7E–G). Low-methylated HGs are accumulated especially in the cell corners (Figure 5G). A similar cellular pattern of localization of low-methylated HG presents in AZ cells 6 h after EPIP application (Figure 7L,M). Low-methylated HGs were less in inactive AZ (Figure 7A,B) than after EPIP application (Figure 7K–M) or naturally active AZ (Figure 7E–G). However, not all of the cell walls of EPIP-treated AZ are labeled (Figure 7K), suggesting that it could be an initial step of cell wall remodeling evoked by the peptide.

In parallel, we performed reactions for high-methylated HG detection. JIM7 labeling is detected in the natural active AZ (Figure 7H–J), EPIP-treated AZ (Figure 7N–P), and inactive AZ (Figure 7C,D). The JIM7 signal in the inactive AZ is stronger (Figure 7C,D) when compared to the reaction with the JIM5 antibody (Figure 7A,B). We also obtained three-dimensional images of the distribution of HGs to better visualize these differences in AZ cells after the EPIP application (see Supplementary Movie S1 for JIM5 and Movie S2 for JIM7). For both epitopes, a strong signal is present in all cell walls. Similar fluorescence is detected in the cell walls of natural active AZ and EPIP-treated AZ. The results of immunofluorescence experiments presented supports that the EPIP treatment induces intensive reorganization of cell wall components in AZ cells.

## 3. Discussion

### 3.1. The AZ Response Evoked by Exogenous EPIP Leads to Abscission Activation

Physiological experiments, as well as those performed on mutants lacking the ability of organ shedding, support that EPIP and PIP are significant peptide molecules involved in signaling pathways that govern separation processes, e.g., flower, fruit, leaf, and floral parts [7,10,14,16,22]. When EPIP is applied to *ida* mutants from *A. thaliana*, the ability of organ abscission is rescued [15]. Furthermore, EPIP has been shown to determine IDA activity in crops, such as citrus, oil palm, poplar, litchi, and importantly, is sufficient to stimulate organ separation in these species [6,7,8]. Thus, this peptide seems to be useful to follow initial steps of separation events, which are crucial for the understanding of regulatory pathways and get the knowledge about primary signaling molecules, which is extremely important for the prevention of premature and excessive organ abscission, particularly in economically important crops [50]. Given that our previous analyses indicated a stimulatory role of EPIP in lupine flower abscission [10], we determined the impact of the EPIP peptide on the IDA–HAE–HSL2 signaling pathway. Firstly, we demonstrated that exogenous EPIP peptide treatments resulted in the upregulation of the expression of downstream components of the IDA signaling system, including *LlHSL* and *LlMPK6* (Figure 1A,B). The peptide treatment also results in the accumulation of LlMPK6 in AZ cells (Figure 2). These results support that EPIP effectively induces abscission, therefore, this experimental approach was useful to perform further analyses to investigate the changes taking place inside the AZ. LlMPK6 localized in floral AZ of lupine (Figure 2C,D), could phosphorylate proteins and activate the signaling pathways of phytohormones, which are essential coordinators of abscission-related processes [51]. As previously shown, a MAP kinase signaling event is also turned on ultimately leading to the induction of PG, cellulases, chitinases, and pectin esterases involved in the execution of the final steps of abscission [23,24,52,53,54].

As the histological analysis showed, the EPIP treatment causes specific changes characteristic for AZ activation. Intensive divisions of AZ cells (Figure 3A–C) might be related to the differentiation enabling the formation of specialized cells that will perform new functions in the active AZ [55]. Cellular divisions accompanied natural abscission of *E. pulcherrima* flowers, *L. angustifolius* cotyledons, and *Castanea* and *Salix* fruits [56,57,58,59]. Intensive cell proliferation, as well as elevated protein content (Figure 3D–F) and many vesicular structures (Figure 3C) in lupine AZ after EPIP treatment, could indicate high metabolic activity of these cells. Vesicle trafficking related to the distribution of cell wall degrading enzymes is an important component of abscission [60]. These enzymes are synthesized de novo to play different functions including (1) middle lamella hydrolysis and cell wall disruption, (2) new cell wall formation between daughter cells, and (3) building of a protective layer on the abscission surface [58,61,62].

### 3.2. The EPIP Treatment Leads to the Disruption of the Redox Balance and Modifies Lipids Composition

Exogenous EPIP peptide affects the redox homeostasis in the flower AZ of yellow lupine (Figure 4 and Figure 5), which is manifested by a rapid (2 h) accumulation of H_2_O_2_ (Figure 5A), preceded by the increased activity of three SOD isoforms (Mn-SOD, Cu/Zn-SOD1, and Cu/Zn-SOD2) (Figure 4). Abscission-accompanied ROS accumulation has been observed in tomato, pepper, as well as lupine [2,13,26,27,63], while the tomato EPIP peptide was recently shown to be involved in ROS homeostasis [64].

SOD are enzymes converting superoxide anion radicals generated by plasma membrane NADPH oxidase and/or in response to distorted photosynthetic and mitochondrial electron transport into H_2_O_2_. This compound is scavenged by CAT and POX [65]. SODs isoforms are characterized by various kinetic proprieties and different gel migration rates, enabling their identification. In the current study, the evidence suggests that the EPIP peptide promotes SOD activity (Figure 4), as well as accelerates H_2_O_2_ production (Figure 5A), consequently leading to the induction of ROS-detoxifying system involving CAT (Figure 5B) and APX (Figure 5B). Immunolocalization confirmed a high abundance of CAT in EPIP treated AZ (Figure 5E–G). Transcriptome analyses carried out in *Manihot esculenta* showed differential expression of genes involved in ROS-induced pathways, encoding, e.g., MeCAT1, MeCu/ZnSOD, confirming the important role of ROS during leaf abscission [28]. Similarly, ethephon-accelerated abscission in the olive tree led to an increase in the transcriptional activity of *OeCAT2* and *OeCAT3* [66]. In chloroplasts, which are strongly modified in yellow lupine AZ following EPIP-induced abscission, APX could be a key component of the H_2_O_2_ detoxification mechanism in the ascorbate-glutathione cycle [67]. Thylakoid-bound isoform (tAPX) and a soluble, stroma-specific isoform (sAPX) of these enzymes catalyze the oxidation of ascorbate [68,69]. The sAPX scavenges the cytosolic H_2_O_2_ and has a higher specificity for phenols as substrates [69]. In yellow lupine, APX activity increases in response to exogenous EPIP (Figure 5B). Thus, it can be supposed that this enzyme participates in chloroplast-related modifications associated with ROS and/or can mediate phenol-dependent oxidation. H_2_O_2_ as a substrate for APX has been shown to take part in the cross-linking reactions between lignin monomers and phenolic residues [70,71]. Specialized lignin structures on the side of the separated organs were shown to be important for the mechanism of cell separation [72]. Our data suggest that APX participates in the plant cell wall loosening process mediated by ROS and/or protective layer formation during EPIP-dependent abscission. Taken together, the EPIP treatment led to the mobilization of antioxidant pathways involving different enzymes active locally in the lupine flower AZ.

The ROS function during the abscission process in lupine could be dual. On the one hand, the influence of unfavorable factors could increase the level of ROS to activate the AZ and initiate organ separation. On the other hand, activation of the AZ leads to ROS formation. In the first case, ROS could act as signal transduction molecules that inform cells about the stress and the necessity for AZ activation and organ abscission. In the second scenario, ROS may play a role as signaling molecules responsible for the coordination of the molecular events accompanying AZ activation, including degradation of lipids, proteins, and nucleic acid [26,73]. Moreover, ROS regulates phytohormonal signal transduction pathways, e.g., ethylene (ET). *Inhibition of ET action downregulates* H_2_O_2_ content in *Erysimum linifolium* petals, which delayed separation [74].

Accumulated ROS may be a signal for changing the fatty acid composition of cell membrane lipids [39,75]. ROS can react with unsaturated FAs of membrane lipids leading to damage of cell membranes and loss of cell turgidity. The OH has been shown to detach the hydrogen from FAs, which initiates the formation of lipid hydroperoxides and lipid peroxidation that consequently increases permeability of cell membranes. Fatty acids are synthesized in plastids, whereas acyl lipid biosynthesis occurs in plastids, ER, and mitochondria. Among acyl lipids, PA is a stress signaling molecule playing a crucial role in degradation, signaling, and lipid turnover (reviewed in [76]). Results obtained here provide evidence that a PA-mediated signal transduction pathway is induced in EPIP-activated flower AZ (Figure 6C). Importantly, PA has been implicated in signaling pathways related to ABA and ET [77,78], which are the main hormonal stimulators of flower abscission in lupine [9], thus a potential relationship between lipid compounds and these phytohormones is strongly suggested. Galactolipids found primarily in the chloroplast (i.e., MGDG and DGDG) were significantly decreased during abscission [39], which implied chloroplast membrane breakdown associated with abscission. It is well known that MGDGs form a single lipid layer highly abundant in thylakoids and inner chloroplast membranes, ensuring greater stability during stress [79]. In contrast, DGDG is more abundant in the outer chloroplast membrane, and the ratio between MGDG and DGDG is regulated by stress conditions [80,81]. In plants, spontaneous lipid (e.g., PC) transport, can be facilitated at membrane contact sites (MCSs) between the ER and outer chloroplast membrane, which promotes the formation of MGDG [82]. In these circumstances, PC is hydrolyzed by PLD to produce PA, which is converted to DAG in the inner chloroplast membrane, and subsequently into MGDG and DGDG [44]. The high level of PC, PLD, and MGDG could support this course of events in floral AZ cells of yellow lupine (Figure 6C,H,I,J,K). A decrease in palmitic acid (16:0) content in response to EPIP (Figure 6B) could be related to changing membrane permeability given that saturated FA C16:0 is used by the cell to regulate membrane fluidity under adverse environmental conditions [83]. In turn, an increase of arachidic acid (20:0; Figure 6B) after EPIP treatment suggests the appearance of stress conditions because this FA is a signaling molecule that modulates plant stress signaling networks [84]. In *Solanaceous*, arachidic acid was shown to act as an elicitor of defense responses and PCD [85,86,87]. EPIP stimulates the accumulation of linoleic acid (LA, 18:2) in acyl lipids of the AZ. Its increased level together with a decreased level of 16:0 in these lipids could affect the membrane fluidity. Moreover, polyunsaturated fatty acids from these lipids could be provided for lipoxygenases (LOXs) or by specific lipases (LIPs). The physiological role of the product of oxidation of 18:2 by LOXs is not known, however, the oxidation (by these enzymes) of α-linolenic acid (18:3) is the first stage of jasmonate (JA) biosynthesis [38,88]. The amount of 18:3 in acyl-lipids of AZ does not change during AZ activation (only a small, insignificant decrease of its relative amount occurred). However, its relative amount in these lipids is high (about 50%), thus only the specific lipase and lipoxygenase activity will be critical factors in proper hydroperoxide production and further synthesis of JAs and derivatives. Jasmonates are hormonal stress factors that could directly regulate changes occurring in AZ cells. JA-dependent mechanism of activating the flower AZ has not been elucidated, thus more detailed studies on specific lipase and lipoxygenase activities in AZ after its activation are planned in our future research on yellow lupine.

AZ activation is associated with cell wall remodeling [89]. Modification of the components forming the cell wall provides direct evidence for the activation and execution of abscission, which is completed by a loosening of the cell wall structure, hydrolysis of the middle lamella, and separation of the organ. Middle lamella strength and stiffness depend precisely on the degree of pectin methylation [90], thus we determined the pectin composition in lupine AZ. Although some studies have examined the cell wall structure prior to, during, as well as after cell separation in different plant species [59,91,92,93], there is no report describing the influence of EPIP on the cell wall-specific changes. Once EPIP is applied and the flower AZ in lupine is activated, we observed a specific distribution pattern of low- (Figure 7K–M) and high-methylated HG (Figure 7N–P), which was similar to those noticed for naturally active AZ (Figure 7E–J). The distribution of methylated pectin (Figure 7K–P) indicates reduced cell wall plasticity and loosening that also suggests a possible contribution of pectin methylesterase in EPIP-dependent abscission.

Previous studies pointed that the pattern of JIM5 and JIM7 epitope distribution changes during the differentiation and activation of the AZ, whereas changes appear to be species-specific. During *Azolla* branch abscission and impatiens leaf abscission, the JIM5 signal appears to decrease, suggesting higher methyl-esterified HG, while during oil palm fruit abscission and the induction of AZ differentiation in the poinsettia leaf base, the JIM5 signal appears to increase suggesting a lower methyl-esterified HG [59,93]. In contrast, no change in JIM5 or JIM7 labeling was observed following flower or fruit abscission in tomato [92]. A comparison of the results obtained in the current study with the available literature data supports the hypothesis that a high level of low or un-methyl-esterified pectin in EPIP-treated and naturally active AZ (Figure 7) may contribute to the abscission-related mechanisms and cell wall loosening, and/or be a part of the defense response and formation of protective scar tissue on the abscised surface after separation. Accumulation of high-methylated pectin detected by JIM7-Ab in lupine AZ (Figure 7J,P) might in turn suggest the secretion of pectin for new cell wall construction of daughter cells formed after divisions.

Briefly, here we show that EPIP influences the downstream elements of the LlIDA pathway—*LlHSL* and MPK6 that leads to cellular changes related to the activation of abscission, and as a consequence floral detachment, as previously shown [10]. The EPIP treatments provoke the disruption of redox homeostasis, which involves the accumulation of H_2_O_2_ and increased activity of the enzymatic antioxidant system. A weakening of the cell wall and membrane structures in response to EPIP application is reflected by pectin demethylation and changes in acyl lipids composition. Furthermore, the EPIP peptide treatment stimulates the appearance of PA, which is a signaling molecule during stress responses. Taken together, we provide support for the role of the EPIP peptide as a small initiator of a range of transformations in AZ cells that lead to flower detachment. Based on presented results, we cannot conclude that EPIP activates each of these processes individually; whether its action is direct, or the activation of one pathway influences another, and then we can talk about the EPIP indirect influence. The time of EPIP-evoked modifications are switching very quickly (2–6 h). So, we cannot assume that all these processes are directly caused by the peptide treatment independently. Nevertheless, we cannot conclude on which process comes first, or the relationship between them. We show induction of the signaling pathway in response to EPIP (HAE-HSL and MPK) within 2 h, so the hypothesis could be that these are the first molecules activated by EPIP, and then the other pathways—redox- and lipids-related are activated leading to flower detachment. A deep analysis of a cause-and-effect relationship between EPIP and different pathways that are switched in AZ is our priority in the immediate future.

## 4. Material and Methods

### 4.1. Plant Material, Growth Conditions, and Treatments

A Taper cultivar of yellow lupine (*Lupinus luteus* L.) was used in this study. Lupines were cultivated under controlled light and temperature conditions as Frankowski et al. [94] described. The flower abscission zone (AZ) located between the pedicel and stem (Supplementary Figure S3A) was excised by using a razor blade under a binocular microscope following our standard procedures [94]. We harvested AZ fragments from several experimental variants: (1) Non-abscised flowers (inactive AZ, IN AZ) (Supplementary Figure S3B); (2) inactive AZ treated with synthetic EPIP peptide (Supplementary Figure S3C); (3) naturally active AZ (active AZ) (Supplementary Figure S3D). Tissue sections (1 mm above and 1 mm below the pedicel-stem junction) were excised in each case.

For the treatment, we used a synthetic peptide (HFSGFLPKRTHMPYSTPSRKHN), which was obtained from the predicted amino acid LlIDL sequence (GeneBank nr AMH85930.1). The peptide was synthesized by Novazym POLSKA s.c. (Poznań, Poland, certificate no. 170302-P013322) and has already been published [10]. Synthetic EPIP solution (100 μM) in 0.05% (*v*/*v*) Tween 20 was applied by small brushes directly onto inactive flower AZs as presented on Supplementary Figure S3C, while inactive AZs were treated with 0.05% Tween 20 solution only [10].

The material was collected at different time variants, which were presented in the results section. Samples used for gene expression analyses (100 mg), lipid profiling (300 mg), or enzymatic assays (500 mg) were frozen in liquid nitrogen and stored at −80 °C. In turn, freshly excised AZ fragments were fixed for ultrastructural assays, as well as histological and immunocytochemical experiments. The experiments were performed in three independent biological replicates.

### 4.2. RNA Extraction and RT-qPCR

ISOLATE II RNA Plant Kit (Bioline) (London, UK) was used for total RNA isolation. Then, 1 μg of RNA primed with anchored oligo (dT)18 was used for the cDNA synthesis with Transcriptor First Strand cDNA Synthesis Kit (ROCHE Diagnostics GmbH, Mannheim, Germany). A Real-Time PCR (RT-qPCR) assay with a LightCycler 2.0 Carousel-Based System (ROCHE Diagnostics GmbH, Mannheim, Germany) and the LightCycler TaqMan Master Kit (ROCHE Diagnostics GmbH, Mannheim, Germany) was used for the *LlHSL*, *LlMPK6*, *LlACT* (reference gene) expression profiling. We followed our standardized qPCR conditions and procedures using gene-specific primers and UPL probes (Supplementary Table S1) [9,11].

### 4.3. Antioxidant Enzymes Activity Determination and H_2_O_2_ Measurements

SOD activity was determined using a method based on nitroblue tetrazolium (NBT) reduction. We applied a modified procedure of Tukaj and Pokora [95], which was optimized already for lupine AZ tissues [96]. Protein content in the supernatant was assayed according to the Bradford method [97]. SODs were visualized as Beauchamp and Fridovich [98] described. The relative SOD isoform activities were normalized to the values obtained for inactive (IN) AZ and expressed as a fold change of the control.

Peroxidase activity assay was performed according to Nakano and Asada [68] with some modifications, which were previously applied for AZs tissues [96]. The presented values correspond to mM pyrogallol oxidized × min^−1^ × mg^−1^ protein.

H_2_O_2_ concentration was analyzed following our previous procedure [96], which is modified protocol of Beers and Sizer [99].

### 4.4. Histological Assays

For microscopy analysis, fresh AZs fragments were fixed, dehydrated, supersaturated, and embedded as previously described [11]. Semithin sections (1 μm) were cut on an Ultracut microtome (Reichert-Jung, Vienna, Austria). The analyses were performed under the LM Zeiss Axioplan microscope (Carl Zeiss, Oberkochen, Germany), equipped with a ProGres C3 digital camera. We used ProGres CapturePro 2.6 software (Jenoptik AG, Jena, Germany).

### 4.5. Immunocytological Assay of MPK6, CAT, JIM5, JIM7, and PLD

The obtained microscopy sections were washed as described previously [9] and then were blocked in 1% bovine serum albumin (BSA) for 2 h. After that, the reactions with primary antibodies were performed. We used primary antibodies provided by Agrisera (Vännäs, Sweden), as follows, anti-MPK6 (AS12 2633), anti-CAT (AS09 501), and anti-PLD (AS09 556) 1:25 in 1% BSA in 1× PBS pH 7.2. Then, a DyLight Alexa 488 conjugated IgG (AS09 633, Agrisera, Vännäs, Sweden) diluted 1:250 in PBS buffer was served as the secondary antibody for 2 h at 37 °C. The control reaction, performed by omitting the incubation with the primary antibody, gave negative results (Supplementary Figure S2B). The nucleic acids were stained with 7 μg/mL 4′,6′-diamidino–2-phenylindole dihydrochloride (DAPI, Sigma-Aldrich, St. Louis, MO, USA) in PBS. For the observations we used an Olympus BX 50 microscope equipped with an Olympus XC50 camera, using a 100× (numerical aperture: 1.4) immersion oil objective.

For the immunolocalization of low- and high-methylated HG, the sections were incubated overnight at 4 °C with JIM5 and JIM7 (PlantProbes, Leeds, UK) diluted 1:20 in PBS buffer, then rinsed in 1× PBS and incubated for 4 h in a goat anti-rat secondary antibody conjugated with FITC (Ab6840, Abcam, Cambridge, UK). In negative control experiments, the primary, secondary, or both antibodies were omitted (Supplementary Figure S2A). After that, the sections were cover-slipped using Mowiol medium and viewed with a fluorescent microscope Leica DM6000 B [100]. For movies, the photos were acquired as Z stacks and deconvolved using 10 iterations of a 3D non-blind algorithm (Autoquant™) to maximize spatial resolution according to the method described by Slazak et al. [100].

Nile Red staining was performed following the modified methodology of Siloto et al. [101] and Greenspan et al. [102]. Sections were stained with 10 µg/mL Nile Red for 5 min and immediately visualized with RHO filter viewed with a fully automated upright fluorescent microscope Leica DM6000.

### 4.6. Lipid Profiling

Total lipids were extracted according to a modified method of Bligh and Dyer [103]. Specifically, AZ fragments were homogenized with a mixture of 3.75 mL of chloroform:methanol (1:2, *v*/*v*) and 1.25 mL of 0.15 M acetic. Then, 1.25 mL of chloroform and 1.25 mL of water was added. The chloroform-lipids fractions were dried under a stream of N_2_ and dissolved in 2 mL of chloroform. Then, they were methylated with 2% H_2_SO_4_ in dry methanol (1 h at 90 °C). After that, methyl-heptadecanoate (17:0-Me, internal standard) was added to the methylation mixtures. Finally, the fatty acid methyl esters were extracted with hexane and analyzed by gas-liquid chromatography (GC-2010; Shimadzu, Kyoto, Japan) on a device equipped with a flame ionization detector and a 60-m × 0.25-mm CP-WAX 58-CB fused-silica column (Agilent Technologies, Santa Clara, CA, USA).

Lipid profiling was performed using a thin-layer chromatography (Merck, Kenilworth, NJ, USA) in chloroform:methanol:acetic acid:water (90:15:10:3, *v*/*v*/*v*/*v*). Visualization of lipid classes was made by short exposure of the plate to iodine vapors. Then, silica gels from areas corresponding to the various lipids were scraped. Lipids were methylated in situ on the gel with 2% H_2_SO_4_ in dry methanol, prepared, and analyzed by gas-liquid chromatography as described above.

### 4.7. Statistical Analysis

Statistical analysis was performed using MS Excel 365 (Microsoft, Redmond, WA, USA) and Statistica 13.1 (StatSoft Inc., Tulsa, OK, USA) software. All data are the results of three biological replicates (one sample was the mix of AZ fragments) with two technical replications (each biological sample was analyzed two times) (*n* = 3). Data were tested for normal distribution and variance homogeneity using Levene’s test. To compare the results obtained for different variants, ANOVA and Tukey’s post hoc test was performed at *p* ≤ 0.05. A two-way ANOVA with treatment variant and time as the two predictor variables was performed (at *p* ≤ 0.05) to evaluate the time-dependent effects of chemicals applied.

## Data Availability

Not applicable.

## Supplementary Materials

The following are available online at https://www.mdpi.com/1422-0067/22/6/3001/s1.
