# Supplementary material for: EPIP-Evoked Modifications of Redox, Lipid, and Pectin Homeostasis in the Abscission Zone of Lupine Flowers
Source: Int J Mol Sci. 2021 Mar 16;22(6):3001. doi: 10.3390/ijms22063001 (PMC7999084; doi:10.3390/ijms22063001)
Supplement: Supplementary file 1 [file ijms-22-03001-s001.zip › Supplementary data.pdf]

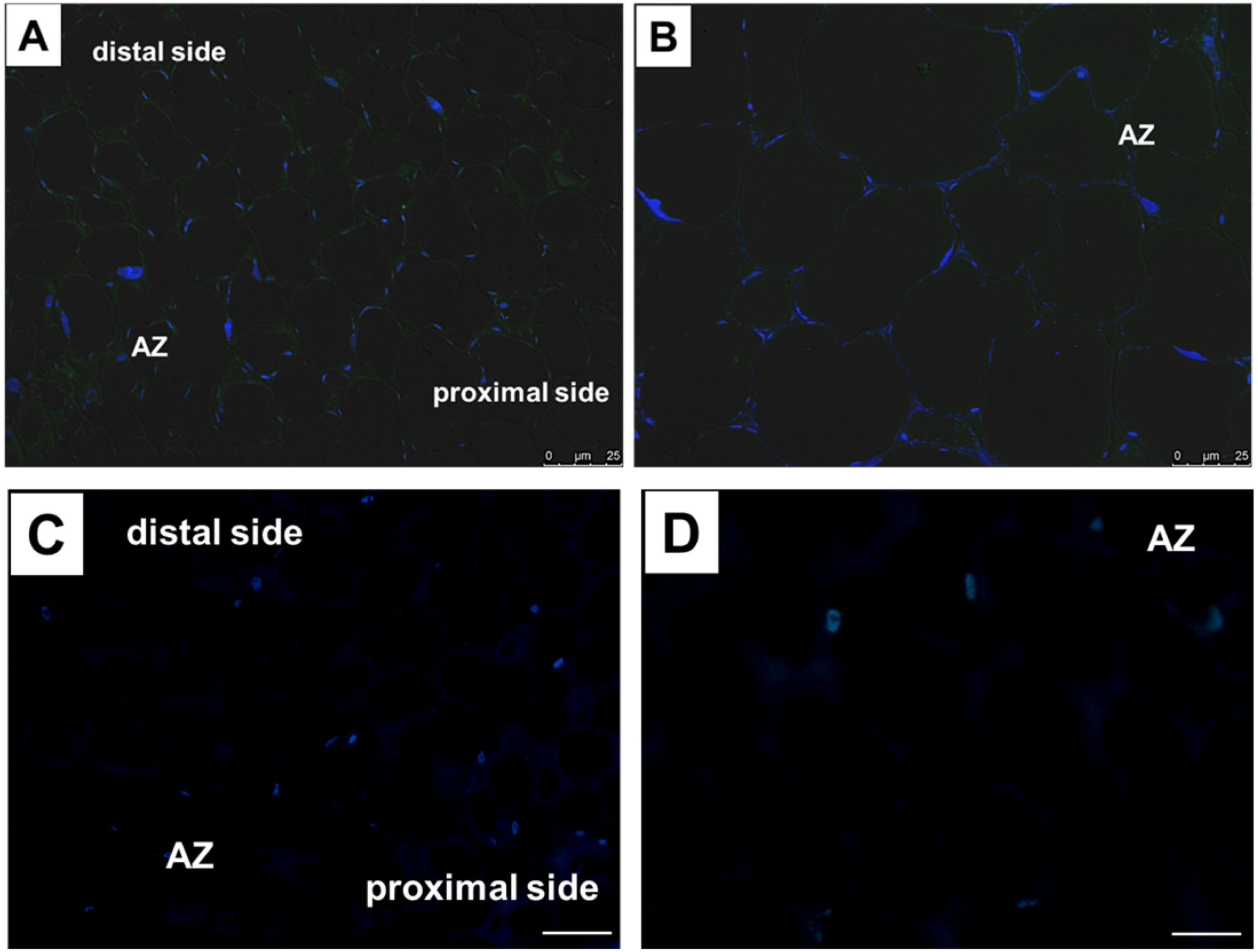

Supplementary Figure S1. The results of a control reaction performer with ommiting primary antibody anti-JIM (**A**) and anti-MPK6, PLD, and CAT (**B**). Control reactions produced negative results compared with those of standard reactions. Nuclei were stained with DAPI. Images A and C present AZ area, B and D show an enlarged view of AZ.

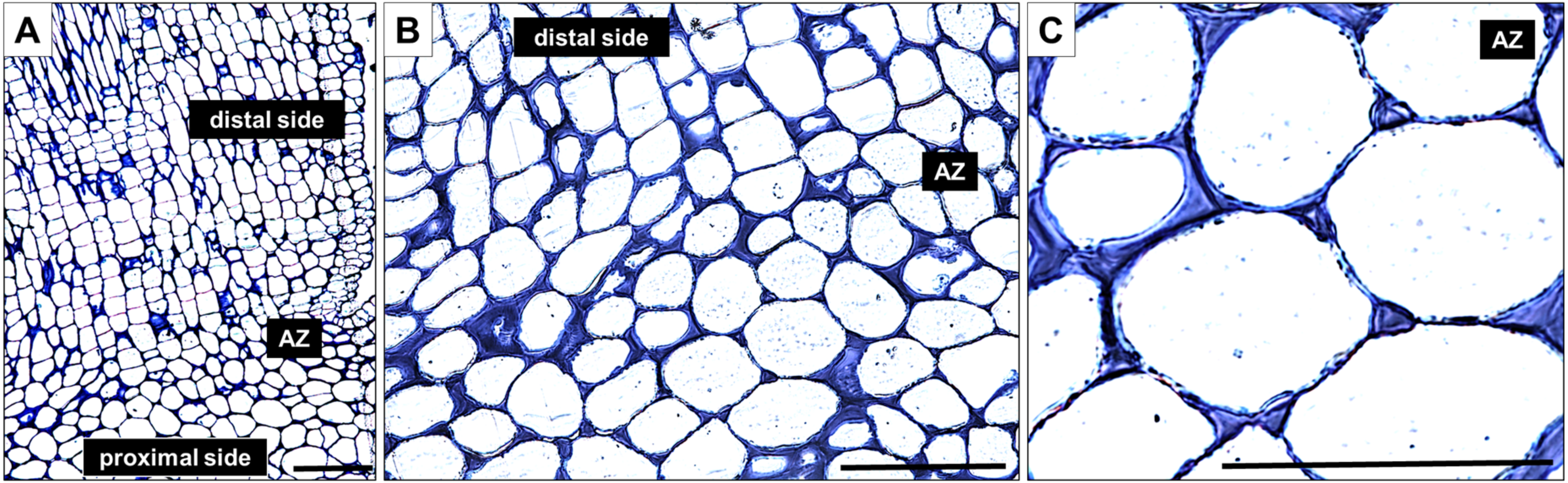

Supplementary Figure S2. Histological analysis of the inactive flower abscission zone (IN AZ) of *Lupinus luteus* L. treated with 0.05% Tween 20 solution. For observations, sections of AZ were collected 24 h after the application. Samples were stained with toluidine blue. Image **B** is an enlarged region of AZ area presented on image A, while image C is a magnification of image B. Scale bars: 70 µm.

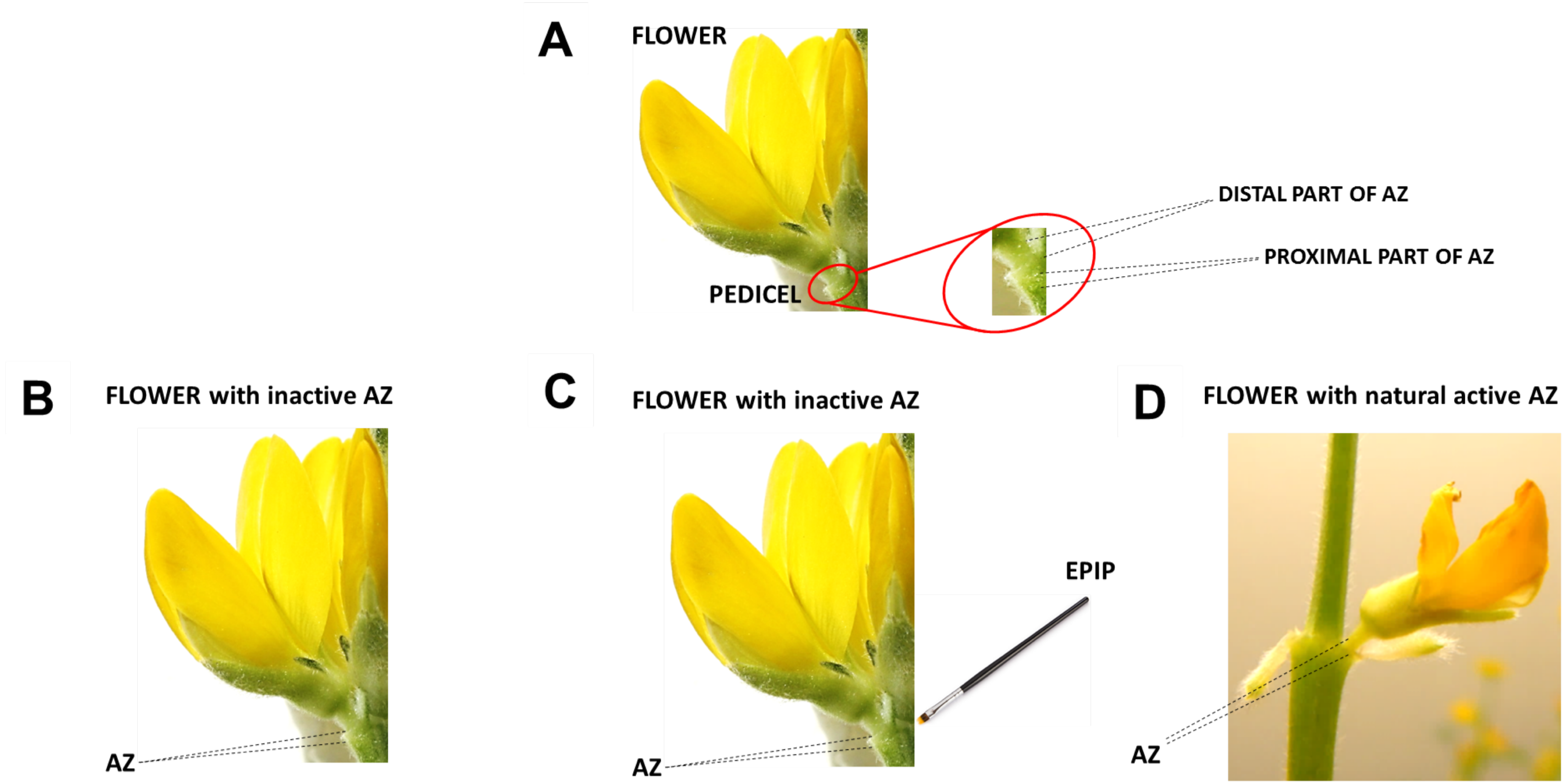

Supplementary Figure S3. *Lupinus luteus* L. flower abscission zone (AZ) is located in the junction of the pedicel and stem (**A**). Non-abscised (**B**), non-abscised and EPIP-treated (**C**), and naturally abscised (**D**) flowers. AZs were collected for analysis.

Supplementary Table 1 Specific primers and probes used in PCR reactions

All primers were synthesized by "Genomed S.A." (Warsaw, Poland).

cDNA of gene	Primer sequence 5'-3'	UPL no.
LlHSL	F: TGATGAAATTGGGTATTTGGGTA<br>R: GAAGCCGGTCAGAAAATTTATTATAG	147
LlMPK6	F: GCTCCTTCGTCATATGGATCA<br>R: AATGTAGACATCATTAAAGCTCTCCTT	147
LlACT	F: TAATGGTTGGGATGGGTCAG<br>R: CAAGGTGAGAATACCCCTCT	165
